# A rare case of melanosis ilei of unclear etiology

**DOI:** 10.1016/j.igie.2025.07.008

**Published:** 2025-07-25

**Authors:** Nikita Parkash, Owen McKay, Ian Simpson, Simon Hew

**Affiliations:** 1 Department of Gastroenterology, Monash Health, Melbourne, Victoria, Australia; 2 Department of Anatomical Pathology, Monash Health, Melbourne, Victoria, Australia

## Description

**Figure 1:**
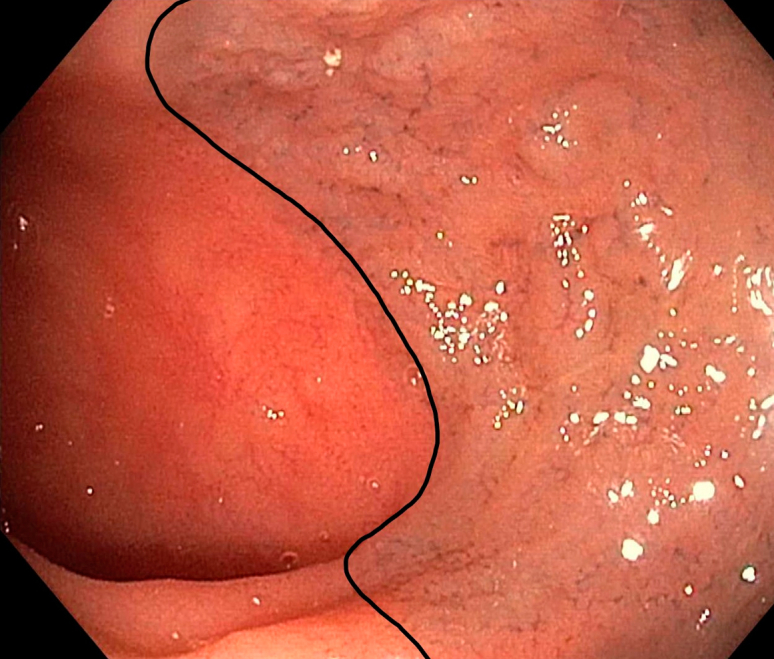
Endoscopic view of terminal ileum with evidence of circumferential gray mucosal discoloration, *outlined in black*.

**Figure 2:**
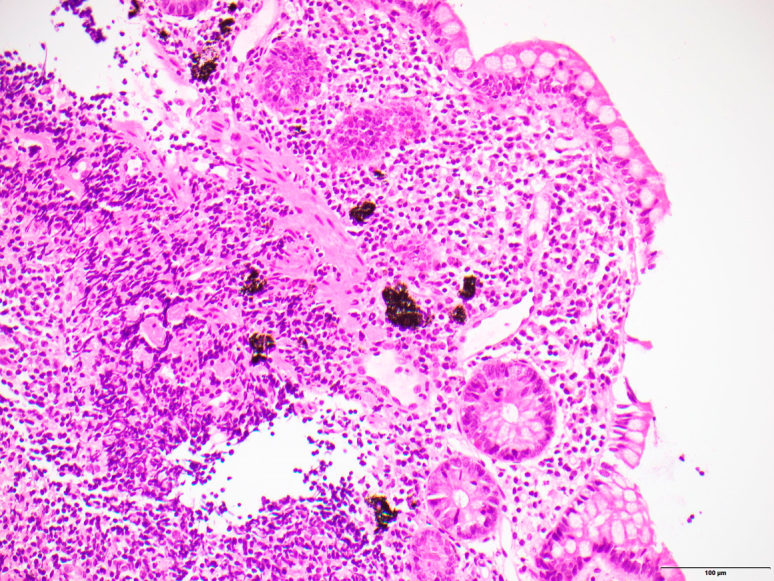
Histopathology slide from terminal ileum biopsy demonstrating dark-brown and black pigmented Peyer’s patches (hematoxylin and eosin, original magnification ×20). The sample was subsequently stained with a Perls’ stain, which was negative.

**Figure 3:**
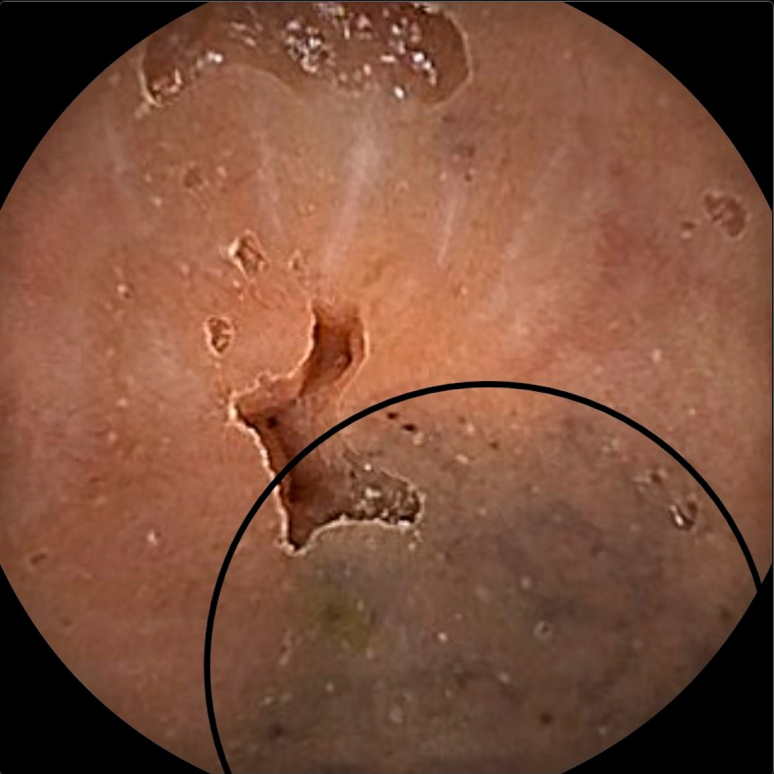
Capsule endoscopy image at 98% transit time, revealing stained small-bowel mucosa, *outlined in black*.

A 47-year-old White man was referred to our gastroenterology outpatient clinic for investigation of recurrent iron deficiency anemia and positive fecal occult blood test results. His medical history included gastroesophageal reflux disease, sleep apnea, hypercholesterolemia, and anxiety. Regular medications were esomeprazole and quetiapine. No oral iron supplementation was taken. On review, the patient denied rectal bleeding, bowel habit changes, or weight loss. His hemoglobin was 110 g/L (reference range 125-175 g/L) and ferritin 7 μg/L (reference range 30-500 μg/L). Gastroscopy was performed, which revealed gastritis and a hiatus hernia with associated esophagitis, and colonoscopy revealed circumferential gray mucosal discoloration in the distal terminal ileum (Fig. 1), but the colon mucosa was normal throughout. Biopsies were performed, and histopathology of the terminal ileum showed dark-brown and black pigmented Peyer’s patches consistent with melanosis ilei (Fig. 2), with a negative Perls’ stain for iron. The patient underwent capsule endoscopy, which similarly revealed stained small-bowel mucosa at 97% to 99% (Fig. 3) transit time, isolated to the distal terminal ileum. The remainder of the bowel was unremarkable. There was no clear cause for the patient's iron deficiency identified endoscopically—melanosis ilei was an incidental finding. However, the hiatus hernia with esophagitis changes, in addition to the gastritis identified during the gastroscopy, could be contributory.

Isolated melanosis ilei is a rare condition typically associated with iron supplementation,^1^^,^^2^ charcoal ingestion,^3^^,^^4^ and chronic laxative use^5^—none of which this patient had consumed. Only a dozen cases of melanosis ilei have been reported,^1^, ^2^, ^3^, ^4^, ^5^ all of which have been attributed to one of the aforementioned causes. The etiology in this patient is unknown—inadvertent ingestion of a compound containing causative agents such as aluminum, magnesium, or silicone may have been possible. The clinical significance of melanosis ilei remains unclear. However, it is thought to be benign,^5^ with mucosal changes resolving after cessation of the offending agent.

## Patient Consent

The patient in this article has given written informed consent to publication of their case details.

## Disclosure

All authors disclosed no financial relationships.
